# Use of whole genome sequencing to determine the genetic basis of visceral myopathies including Prune Belly syndrome

**DOI:** 10.1007/s44162-023-00012-z

**Published:** 2023-06-05

**Authors:** Robert M. Geraghty, Sarah Orr, Eric Olinger, Ruxandra Neatu, Miguel Barroso-Gil, Holly Mabillard, Genomics England Research Consortium, Ian Wilson, John A. Sayer

**Affiliations:** 1 Renal Services, The Newcastle Upon Tyne Hospitals NHS Foundation Trust, Freeman Road, Newcastle Upon Tyne, NE7 7DN UK; 2 Faculty of Medical Sciences, Translational and Clinical Institute, Newcastle University, Central Parkway, Newcastle Upon Tyne, NE1 3BZ UK; 3 Faculty of Medical Sciences, Biosciences Institute, Newcastle University, Central Parkway, Newcastle Upon Tyne, NE1 3BZ UK; 4 National Institute for Health Research Newcastle Biomedical Research Centre, Newcastle Upon Tyne, NE4 5PL UK

**Keywords:** Visceral myopathy, Prune Belly syndrome, *ACTG2*, *MYH11*, Whole genome sequencing

## Abstract

**Objectives/aims:**

The visceral myopathies (VM) are a group of disorders characterised by poorly contractile or acontractile smooth muscle. They manifest in both the GI and GU tracts, ranging from megacystis to Prune Belly syndrome. We aimed to apply a bespoke virtual genetic panel and describe novel variants associated with this condition using whole genome sequencing data within the Genomics England 100,000 Genomes Project.

**Methods:**

We screened the Genomics England 100,000 Genomes Project rare diseases database for patients with VM-related phenotypes. These patients were screened for sequence variants and copy number variants (CNV) in *ACTG2*, *ACTA2*, *MYH11*, *MYLK*, *LMOD1*, *CHRM3*, *MYL9*, *FLNA* and *KNCMA1* by analysing whole genome sequencing data. The identified variants were analysed using variant effect predictor online tool, and any possible segregation in other family members and novel missense mutations was modelled using in silico tools. The VM cohort was also used to perform a genome-wide variant burden test in order to identify confirm gene associations in this cohort.

**Results:**

We identified 76 patients with phenotypes consistent with a diagnosis of VM. The range of presentations included megacystis/microcolon hypoperistalsis syndrome, Prune Belly syndrome and chronic intestinal pseudo-obstruction. Of the patients in whom we identified heterozygous *ACTG2* variants, 7 had likely pathogenic variants including 1 novel likely pathogenic allele. There were 4 patients in whom we identified a heterozygous *MYH11* variant of uncertain significance which leads to a frameshift and a predicted protein elongation. We identified one family in whom we found a heterozygous variant of uncertain significance in *KCNMA1* which in silico models predicted to be disease causing and may explain the VM phenotype seen. We did not find any CNV changes in known genes leading to VM-related disease phenotypes. In this phenotype selected cohort, *ACTG2* is the largest monogenic cause of VM-related disease accounting for 9% of the cohort, supported by a variant burden test approach, which identified *ACTG2* variants as the largest contributor to VM-related phenotypes.

**Conclusions:**

VM are a group of disorders that are not easily classified and may be given different diagnostic labels depending on their phenotype. Molecular genetic analysis of these patients is valuable as it allows precise diagnosis and aids understanding of the underlying disease manifestations. We identified *ACTG2* as the most frequent genetic cause of VM. We recommend a nomenclature change to ‘autosomal dominant ACTG2 visceral myopathy’ for patients with pathogenic variants in *ACTG2* and associated VM phenotype*s*.

**Supplementary Information:**

The online version contains supplementary material available at 10.1007/s44162-023-00012-z.

## Introduction

Visceral myopathies (VM) (OMIM 155310) are a group of rare congenital conditions of smooth muscle dysfunction. They are characterised by variable phenotypes, ranging from megacystis (massively distended bladder) and severe feeding intolerance secondary to intestinal dysmotility at the most severe end of the spectrum, to intermittent abdominal distension and functional intestinal obstruction at the milder end of the spectrum. These visceral myopathies have been grouped into three distinct phenotypes: megacystis-microcolon intestinal hypoperistalsis syndrome (MMIHS) (OMIM 619362, 249,210, 619,365, 619,431, 619,351), chronic intestinal pseudo-obstruction (CIPO) (OMIM 3000048) and Prune Belly syndrome (PBS) (OMIM 100100).

The clinical consequences of patients with these groups of phenotypes can be significant [17]. Those with MMIHS often require a long-term indwelling urinary catheter. This is not a permanent solution, and often, surgical intervention to create a vesicostomy is undertaken [16, 53]. The intestinal manifestations of MMIHS are often more significant, ultimately requiring surgical intervention to form a gastrostomy for feeding or ileostomy for faecal diversion. Failing these interventions, patients often require parenteral nutrition (PN), with the associated long-term risks and those of a permanent central line. Mortality in these patients is over 40% within the first year [53].

Given the rarity and broad phenotypic range of these diseases, a true epidemiologic picture is difficult to attain. CIPO is the only phenotype to have been studied in detail, with a study in Japanese babies estimating the incidence at 0.21 (male) and 0.24 (female) cases per 100,000 population [17]. As a less severe phenotype, CIPO is associated with a longer life expectancy. However, these patients are again often managed surgically with gastrostomies or surgical jejunostomies and PN [25]. One study reported survival at 10 years following initiation of PN at 75% in adults [1].

To summarise the clinical picture, VM presents with a range of phenotypes, most requiring surgical intervention for feeding, urinary or faecal diversion and with a variable mortality rate. Research into the underlying molecular genetic causes of visceral myopathy has identified several genes in which pathogenic variants have been associated with the disease. Heterozygous pathogenic variants in *ACTG2* (which encodes a smooth muscle actin) were initially identified by Wangler et al. and subsequently confirmed in other clinical studies [2, 20, 32, 52]. In murine expression studies of *Actg2*, there was evidence of high expression in the bladder and bowel [34, 44]. Unfortunately, there have been no murine knock-out studies examining *Actg2* in more detail.

Aside from *ACTG2*, other genes underlying visceral myopathies have been described. These include *ACTA2*, encoding a smooth muscle actin protein found in bladder/bowel but predominantly in vascular tissue [34]. *ACTA2* variants inherited in an autosomal dominant fashion give rise to megacystis and variably, intestinal malrotation and hypoperistalsis [35]. However, smooth muscle actins encoded by this gene are also associated with vascular and ciliary smooth muscles, and therefore, aneurysms and mydriasis complete this phenotypic picture. There are several other genetic causes such as *MYH11* (encoding a myosin-heavy chain) [8], which may be inherited in either an autosomal dominant or recessive manner. Phenotypes associated with *MYH11* disease-causing variants include CIPO and chronic gut motility disorders inherited as an autosomal dominant pattern [5, 9]. Biallelic variants in *MYH11* have been reported to cause the more severe MMIHS phenotype [51].

The remainder is inherited in an autosomal recessive manner including *MYLK* (encoding a myosin-light chain kinase) [11], *LMOD1* (encoding leiomyodin) [13], *MYL9* (encoding a regulatory myosin light chain) [37], and *FLNA* (encoding Filamin A — an actin-binding protein) [19]. Finally, there are several genes associated with intestinal hypoperistalsis but are due to mitochondrial disorders, rather than inherited myopathies. These include the following: *EDNRB*, *EDN3*, *SOX10*, *SGOL1*, *RAD21* and L1CAM [2].

Whole genome sequencing (WGS) is becoming commonly applied to rare diseases in order to define its underlying molecular basis [48]. We utilised WGS data within the Genomics England 100,000 Genomes Project to determine the molecular basis of 76 patients recruited with a VM-related phenotype.

## Methods and materials

### Participants

Participants with suspected visceral myopathy disorders were recruited to the 100,000 Genomes.

Project (main programme) between 2015 and 2018 with megacystis-microcolon intestinal hypoperistalsis syndrome (MMIHS), chronic intestinal pseudo-obstruction (CIPO) or Prune Belly syndrome (PBS). All participants provided written informed consent, and the study was approved by the HRA Committee East of England Cambridge South (REC Ref 14/EE/1112).

### Clinical information

Human Phenotype Ontology (HPO) terms were used to classify clinical features by organ system using a branching tree incorporating increasing levels of detail [22] (Supplementary Table S1). Patients were assigned to a disease domain, and this allowed the application of a virtual gene panel to the genomic data, based on known disease genes associated with the phenotype.

### Whole genome sequencing

DNA extraction, quantification and sequencing were performed according to a national specification (Illumina TruSeq, HiSeq 2500 and HiSeq X) [48] with reads aligned to the Genome Reference Consortium Human Genome Build 38 (GRCh38) for the earlier participants recruited and GRCh38 for later participants using Isaac Genome Alignment Software. Family-based variant calling of single-nucleotide variants and insertion-deletions for chromosomes 1–22 and X was performed using the Platypus variant caller [40].

### Variant analysis

Genomes were analysed in families, and variants were classified into four ‘tier’ groups according to the probability of the variant being causative [43]. Tier 1 included loss-of-function variants and de novo missense or splice region variants in genes on the virtual gene panels applied, tier 2 included missense and splice region variants in genes on the virtual gene panels applied, tier 3 included other rare variants, and a final group of unclassified variants had higher population frequency, or the segregation pattern in the family was not consistent with phenotypic information available. Virtual gene panels were chosen according to each participant’s phenotypes, using curated ‘PanelApp’ gene lists [30], to prioritise variants likely to be causative and to minimise ‘incidental findings’. There was no specific gene panel for VM; therefore, a bespoke virtual gene panel consisting of *ACTG2*, *ACTA2*, *MYH11*, *MYLK*, *LMOD1*, *CHRM3*, *MYL9*, *FLNA* and *KCNMA1* was applied. Tiers 1–3 gene variants were accessed from the Main Programme v11_2019-11–28. All tiered gene variants had passed in-house Genomics England quality control. Sequence variants were also prioritised using Exomiser [42]. Variants classified as pathogenic, likely pathogenic or pathogenic/likely pathogenic were identified using ClinVar [24] for GRCh38 and GRCh37 and compared against tier 1–3 variants using bedtools intersect (https://bedtools.readthedocs.io). If parental data were available, a segregation of alleles and phenotypes was performed to determine if alleles were inherited or de novo.

### Clinical review

Tier 1 and tier 2 variants, the top ten prioritised variants by Exomiser and ClinVar pathogenic/likely pathogenic variants, were reviewed by a clinical geneticist and classified by ACMG criteria [39], using information from gnomAD [21], Ensembl [56], VarSome [23], OMIM and review of the literature. Feedback from the GMC laboratories and clinical teams was incorporated when available. Variant quality was checked using the Integrative Genomics Viewer (IGV) [41]. The molecular diagnosis was described as ‘definite’, ‘probable’ or ‘possible’ based on the ACMG classification of the variant(s), the inheritance pattern and the clinical fit between the patient’s HPO terms and the reported clinical phenotypes for the genetic variant. The contribution was described as ‘full’ or ‘partial’ depending on whether the whole phenotype or only one aspect could be explained by the identified variant(s) from the VM virtual gene panel.

### Genome-wide variant burden tests

Individuals with a VM-related phenotype were contrasted with a control group of individuals. Controls were selected from the rare diseases cohort of individuals and were (i) not probands, (ii) labelled ‘unaffected’ and had no HPO terms that included any of the words or partial words: ‘bladder’, ‘ureter’, ‘urin’, ‘renal’ or ‘muscl’. A merged control vcf file was produced using bcftools. The genes and gene locations considered were from Ensembl gene coordinates version 96 for GRCh38. These genes were extracted using tabix and annotated using variant effect predictor (VEP) [31]. The number of variants per gene per individual that was of (i) of frequency < 0.001 and (ii) could have high and moderate impact consequences (frameshift, missense and stop variant annotations) was counted using a custom R script, and, for each gene, the number of individuals with at least one qualifying variant was compared between cases and controls using a Fisher’s exact test.

### Copy number variant analysis

Copy number depth was calculated using bcftools depth every 20 bases for the panel genes and for control genes in three sets (20 neighbouring, 20 more distant on the same chromosome and 20 on different autosomes). The average depth for each individual and for each gene in the panel was scaled by the average depth on the control genes for each control set. A copy number was then called based on the relative depth of each gene to the control set. Differences between calls based on neighbouring control sets and far control sets are investigated further for larger deletions or duplications [7, 18].

### Spatial modelling

The spatial structure of KCNMA1 proteins was modelled and visualised with AlphaFold-2 and PyMOL 2.3 software to determine protein folding of missense variant of interest [47].

### Phenotype enrichment analysis

In an unbiased approach, the application of a gene variant analysis of *ACTG2* to WGS data from all probands and relatives (*n* = 65,507, including all phenotypes) in the Genomics England 100,000 Genomes Project Rare Disease project who carried out. Results were annotated and then filtered for probands alone and missense variants in *ACTG2* with an *AF* < 1% (in any gnomAD population) and a CADD score > 20 or synonymous *ACTG2* variants with an *AF* < 1% (in any gnomAD population). The phenotypes of probands with *ACTG2* variants (either missense or synonymous as a control group) were analysed for associated ICD10 terms. The prevalence of each ICD10 term that was encountered was calculated and phenotype enrichment ratio calculated. Fisher’s exact test was computed for each retained analysed phenotype and phenotypes ranked according to *p*-value of statistical significant enrichment.

## Results

**Fig. 1:**
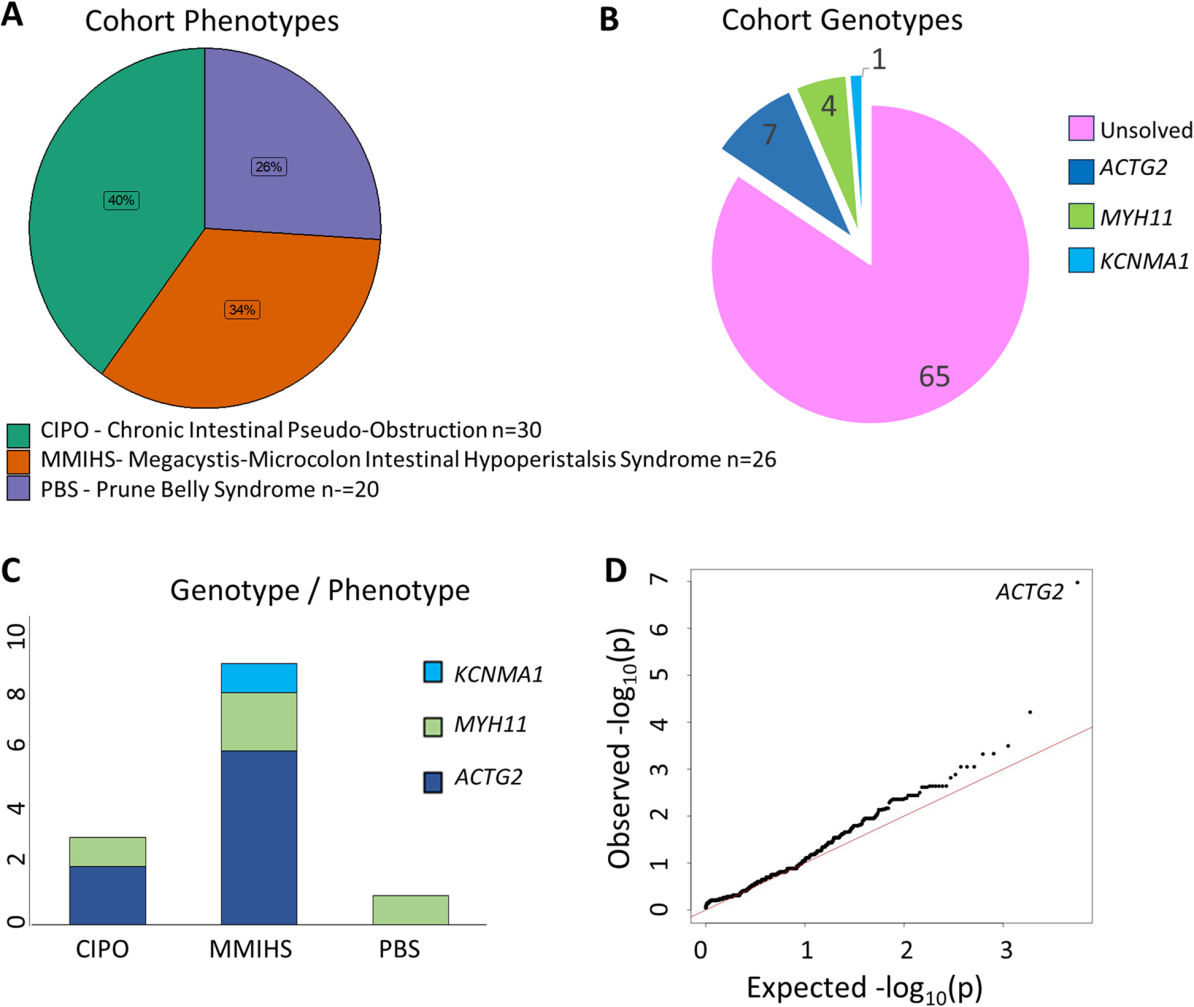
Visceral myopathy cohort and genetic variants identified in this study. **A** Cohort of 76 patients with visceral myopathy phenotypes including sub-groups CIPO, MMIHS and PBS. **B** Patients genotype with pathogenic/likely pathogenic variants in ACTG2; VUS in MYH11 and VUS in KCNMA1. **C** Genotype–phenotype correlations within the identified patients. **D** Quantile–quantile plot for rare variants associated with visceral myopathy phenotypes following genome-wide variant burden test. Shown are empirically observed quantiles of rare gene effects (AF < 0.001) (y-axis) as a function of quantiles expected from a normal distribution with the same mean and variance as the empirical distribution (x-axis). Variants in ACTG2 were the only statistically significant finding (*p* = 1.1 × 10^−^.^7^)

Overall, there were 76 patients in the Genomics England 100,000 Genomes Project rare disease cohort with phenotypes that represented VM phenotypes (*n* = 30 with CIPO, *n* = 26 with MMIHS and *n* = 20 with PBS) (Fig. 1A). Application of the custom VM gene panel demonstrated no participants with pathogenic or likely pathogenic variants in *ACTA2*, *LMOD1* or *MYL9*. No rare CNVs within the virtual gene panel were detected.

In total, we identified 11 VM patients with rare *ACTG2* variants, 6 with rare *MYH11* variants, 3 with rare *MYLK* variants, 1 with a rare *CHRM3* variant, 3 with rare *FLNA* variants and 1 with a rare *KCNMA1* variant of uncertain significance. In order to determine the pathogenicity, an in silico analysis of each of these genetic findings was performed. For each patient, the clinical phenotypes and any genetic segregation analysis where available were also reviewed to determine which patients with VM-associated phenotypes could be genetically solved.

### Patients with rare ACTG2 alleles

Heterozygous mutations in *ACTG2* are associated with VM phenotypes including MMIHS type 5 (OMIM 619431) and visceral myopathy type 1 (OMIM 155310). We identified 11 VM patients carrying rare *ACTG2* alleles, 9 of whom had severe early onset VM phenotypes. Of these 11 patients, 5 were determined to be genetically ‘solved’ by Genomics England with a diagnostic variant in *ACTG2* reported in the Genomics England exit questionnaire. These variants included known pathogenic missense alleles p.Arg40His (patient 1), p.Arg178Leu (patient 3), p.Arg257Cys (patient 4, de novo) and p.Arg257His (patient 5, de novo; patient 6, de novo). In another VM patient (patient 2), we identified a previously reported and known pathogenic allele p.Arg148Cys, inherited from their affected father allowing this patient to be genetically solved (Table 1 and Supplementary Fig. S1). For the remaining 5 patients with VM phenotypes, who were unsolved by Genomics England as segregation data for potential causative variants was lacking, we identified novel heterozygous *ACTG2* variants (Table 1). Pathogenicity scores suggested that one of these alleles was likely pathogenic and disease causing (c.338C > T; p.Pro113Leu, patient 9), although family segregation data is incomplete, whilst 4 were classified as variants of uncertain significance (VUS). Heterozygous pathogenic and likely pathogenic variants (all missense alleles) in *ACTG2* therefore solved 7 out of 76 (9.2%) VM patients and were associated with phenotypes related to CIPO and MMIHS (Fig. 1B, C).

**Table 1:** Patient phenotypes and *ACTG2* alleles identified following WGS

Participant	Ethnicity	Phenotype	*ACTG2* nucleotide change	*ACTG2* amino acid change	ACMG classification	SIFT score	PolyPhen-2 score	Allele frequency	Solved by GEL	Comments	References
1 — female	White, British	Visceral myopathy, abdominal distension, abdominal pain, foetal megacystis, gastrointestinal dysmotility, constipation, intestinal malrotation, intestinal pseudo-obstruction	**c.119G > A**	p.(Arg40His)	Pathogenic (11 points) P, PP5, PM1, PM5, PP3, PM2	Deleterious (0.02)	Probably damaging (0.991)	0.00001 (PAGE study [4]	Y	rs587777386 Unaffected mother wild type genotype; unaffected father, no genotype	[2, 32, 36, 38, 52, 55]
2 — male	White, European	Intestinal pseudo-obstruction, gastrointestinal dysmotility, constipation	**c.442C > T**	p.(Arg148Cys)	Pathogenic (10 points) P, PM5, PP3, PM1, PP5, PM2	Deleterious (0.02)	Probably damaging (0.989)	0.000004 (Gnomad)	*Y*	rs587777383 AD pattern, affected father also has pathogenic allele	[15, 26, 38, 39]
3 — female	White, European	Pseudo-obstruction, microcolon, megacystis, hypoperistalsis, feeding difficulties	**c.533G > T**	p.(Arg178Leu)	Likely pathogenic (17 points) LP, PS3, PM5, PP3, PP5, PM2, PP2	Deleterious (0)	Probably damaging (0.775)	N/A	*Y*	rs587777384 Unaffected mother, wild-type genotype; unaffected father no genotype	[6, 33, 46, 49, 52]
4 — male	N/A	Intestinal pseudo-obstruction, myopathy, feeding difficulties, gastrointestinal dysmotility, megacystis, congenital hydronephrosis	**c.769C > T**	p.(Arg257Cys)	Pathogenic (16 points) P, PP5, PP3, PM5, PM2, PP2	Deleterious (0.04)	Probably damaging (0.989	0.00008 (PAGE study [4])	*Y*	rs587777387 De novo, both parents unaffected and wild type	[2, 12, 28, 29, 32, 33, 36, 38, 39, 46, 49, 52, 54]
5 — female	N/A	Intestinal pseudo-obstruction, myopathy, feeding difficulties, gastrointestinal dysmotility, megacystis, constipation, neuromuscular dysfunction of bladder	**c.770G > A**	p.(Arg257His)	Pathogenic (16 points) P, PP5, PP3, PM5, PM2, PP2	Deleterious (0.04)	Probably damaging (0.989)	N/A	*Y*	rs797044959 De novo both parents unaffected and wild type	[33, 46, 49, 52]
6 — male	N/A	Intestinal pseudo-obstruction, myopathy, feeding difficulties, gastrointestinal dysmotility	**c.770G > A**	p.(Arg257His)	Pathogenic (16 points) P, PP5, PP3, PM5, PM2, PP2	Deleterious (0.04)	Probably damaging (0.989)	N/A	*Y*	rs797044959 De novo both parents unaffected and wild type	[33, 46, 49, 52]
7 — male	White, British	Intestinal pseudo-obstruction, microcolon, megacystis	**c.226A > T**	p.(Ile76Phe)	Uncertain significance (3 points) VUS, PP3, PM2, PP2	Deleterious (0)	Probably damaging (0.961)	N/A	*N*	Novel unaffected mother, wild-type genotype; unaffected father no genotype	Novel
8 — female	Asian	CAKUT, bladder extrophy	**c.287G > A**	p.(Arg96His)	Uncertain significance (4 points) VUS, PP3, PM2, PP2	Deleterious (0.04)	Benign (0.219)	9.02 × 10^−4^ (Genomics England)	*N*	rs1363649764 — VUS Unaffected parents, no genotype	Novel
9 — female	White, British	Megacystis, hydroureter, hydronephrosis, intestinal malrotation, constipation, microcolon	**c.338C > T**	p.(Pro113Leu)	Likely pathogenic (6 points) LP, PP3, PM1, PM5, PM2	Deleterious (0.01)	Probably damaging (1)	N/A	*N*	Novel unaffected mother, wild-type genotype; unaffected father, no genotype	Novel
10 — female	White, British	Cystic kidney disease, hypertension, recurrent UTIs	**c.386A > G**	p.(Asn129Ser)	Uncertain significance (2 points) VUS, PM2, PP2	Tolerated (0.14)	Benign (0.04)	0.0045 (Genomics England)	*N*	rs77469596 Father affected, no genotype; unaffected mother, no genotype	Novel
11 — male	White, British	Prune Belly syndrome, congenital hydronephrosis	**c.850A > G**	p.(Met284Val)	Uncertain significance (4 points) VUS, PP3, PM2, PP2	Deleterious (0.01)	Benign (0.382)	N/A	*N*	Novel unaffected mother, no genotype; unaffected father, no genotype	Novel

*ACTG2* NM_001199893. SIFT values of between 0 and 0.05 are predicted to affect protein function. PolyPhen-2 values between 0.85 and 1.0 are confidently predicted to be damaging, values between 0.15 and 1.0 range are possibly damaging and values between 0.0 and 0.15 are predicted to be benign

### Patients with rare MYH11 alleles

Both heterozygous variants [5, 9] and biallelic variants [51] in *MYH11* are typically associated with VM phenotypes that include autosomal recessive MMIHS type 2 (OMIM 619351) and autosomal dominant visceral myopathy type 2 (OMIM 619350). Biallelic variants typically cause more severe clinical phenotypes, suggesting a spectrum of disease according to inheritance pattern. In our cohort, we identified 6 VM patients with heterozygous *MYH11* variants, none of whom was previously genetically solved by Genomics England. There was one participant (patient 16), with Prune Belly syndrome with a novel *MYH11* heterozygous missense variant (p.(Lys1141Gln)), classified as a VUS and unlikely on its own to explain this severe phenotype. There were four patients (patients 12, 13, 14, 15), with variable clinical phenotypes of VM, who had the same heterozygous *MYH11* c.5819del; p.(Pro1940Hisfs*91) frameshift allele (Table 2 and Supplementary Fig. S2). This variant is classified as a VUS and was found in patient 15 with a mild phenotype with features of constipation, colostomy and urinary retention (Table 2). This *MYH11* frameshift allele is identical to the *MYH11* allele reported by Gilbert et al. in a three-generation family with five affected individuals, with the proband diagnosed with constipation in infancy and followed but infantile pseudo-obstruction at the age of 11 years. Anal manometry measurements performed at 20 years of age showed findings consistent with a smooth muscle myopathy [9]. Interestingly, of 2 of the 4 patients with this identical allele also presented with intestinal pseudo-obstruction. The same allele was also reported by Dong et al. in a three-generation family with 7 affected with a CIPO phenotype, including 3 individuals with bowel complications including bowel obstruction, rectal prolapse and malrotation of the bowel [5]. Together, these cases provide an accumulation of evidence for a role in VM phenotypes.

**Table 2:** Patient phenotypes and *MYH11* alleles identified following WGS

Participant	Ethnicity	Phenotype	*MYH11* nucleotide change	*MYH11* amino acid change	ACMG classification	SIFT score	PolyPhen-2 score	Allele frequency	Solved by GEL	Comments	Reference
11 — male	White, British	PBS, congenital hydronephrosis	**c.3766A > C**	p.(Lys1256Gln)	Likely benign (− 2 points) BP4, BP1, PM2	Tolerated (0.51)	Probably damaging (0.93)	0.000299 (gnomAD)	*N*	rs149241435 Mother and father unaffected, no genotype available	Novel
12 — male	White, British	CAKUT, megacystis, hydronephrosis, recurrent UTIs	**c.5819del**	p.(Pro1940Hisfs*91)	Uncertain significance (2 points)	Deleterious (0.02)	Probably damaging (0.991)	6.82 × 10^−5^ (gnomAD)	N	rs747392139 Mother unaffected, genotype wild type; father unaffected, no genotype available	[5, 9]
13 — female	White, British	Intestinal pseudo-obstruction, myopathy	**c.5819del**	p.(Pro1940Hisfs*91)	Uncertain significance (2 points)	Deleterious (0.02)	Probably damaging (0.991)	6.82 × 10^−5^ (gnomAD)	*N*	rs747392139 Mother unaffected, no genotype available; father affected, no genotype available	[5, 9]
14 — male	White, British	Intestinal pseudo-obstruction, megacystis, myopathy, constipation, gastrointestinal dysmotility	**c.5819del**	p.(Pro1940Hisfs*91)	Uncertain significance (2 points)	Deleterious (0.02)	Probably damaging (0.991)	6.82 × 10^−5^ (gnomAD)	*N*	rs747392139 Mother affected, no genotype available; father unaffected, no genotype available	[5, 9]
15 — male	White, British	PBS, retention of urine, colostomy	**c.5819del**	p.(Pro1940Hisfs*91)	Uncertain significance (2 points)	Deleterious (0.02)	Probably damaging (0.991)	6.82 × 10^−5^ (gnomAD)	N	rs747392139 Mother and father unaffected, no genotype available	[5, 9]
16 — male	White, British	Hypertension, chronic kidney disease	**c.3421A > C**	p.(Lys1141Gln)	Uncertain significance (0 point) PM2, BP1	Deleterious (0.01)	Probably damaging (0.88)	N/A	*N*	rs797045725 Mother and father unaffected, no genotype available	Novel

*MYH11* NM_022844. SIFT values of between 0 and 0.05 are predicted to affect protein function. PolyPhen-2 values between 0.85 and 1.0 are confidently predicted to be damaging, values between 0.15 and 1.0 range are possibly damaging and values between 0.0 and 0.15 are predicted to be benign

Patient 11, with a Prune Belly syndrome phenotype, had a *MYH11* likely benign allele in addition to the identified *ACTG2* VUS allele c.850A > G; p.(Met284Val) which is insufficient evidence to solve this case. In total, heterozygous predicted loss-of-function variants in *MYH11* but classified as VUS by ACMG criteria (Table 2) were found in 4 (patients 12, 13, 14, 15) out of 76 (5%) patients with VM phenotypes (Fig. 1B).

### Patients with rare MYLK alleles

Biallelic variants in *MYLK* are associated with VM phenotypes that include MMIHS type 1 (OMIM 249210). In our VM cohort, we identified 3 VM patients with variable phenotypes with heterozygous *MYLK* variants (Supplementary Table S2). The first variant was a synonymous variant, and the second was a missense variant. The SpliceAI results of the synonymous variant were low and is therefore likely compensated, and the allele is likely benign. The missense variant was predicted as likely benign. Given that known *MYLK* genetic variants associated with VM are all biallelic (homozygous frameshift mutations), these variants are unlikely to be pathogenic and causative of the disease phenotype in these VM cases.

### Patients with rare CHRM3 alleles

Biallelic variants in *CHRM3* are associated with Prune Belly syndrome (OMIM 100100). We identified one VM patient (patient 20) with a heterozygous missense *CHRM3* variant, classified as a VUS (Supplementary Table S3). This heterozygous variant alone is unlikely to be pathogenic and causative of the disease phenotype in this case.

### Patients with rare FLNA alleles

Pathogenic variants in *FLNA* cause an X-linked recessive form of neuronal intestinal pseudo-obstruction (OMIM 300048). We identified one male participant with *FLNA* variants. This participant (patient 21) had 3 *FLNA* rare variants, 2 splice region variants and 1 synonymous variant. The splice region variants identified were c.5741-8C > T and c.5289 + 4C > T and predicted to be of low impact. The SpliceAI results of the synonymous variant were also low and is therefore likely compensated suggesting these variants were unlikely to be causative of the disease phenotype in this case (Supplementary Table S4).

### Genome-wide variant burden test

We performed a genome-wide variant burden test using our cohort of VM patients as our target population and with 918 selected controls. There was only one highly significant gene identified (*ACTG2* (*p* = 1.1 × 10^−7^)) from the assembled alleles (Fig. 1D and Supplementary Table S5). The finding of *ACTG2* as the most significant associated gene with VM phenotypes is consistent with our findings from the application of a virtual gene panel for this cohort. It also suggests that other genetic causes of VM within this cohort are rare.

### Phenotype enrichment analysis

Up to this point, our investigations had been limited to a well-defined cohort of patients with defined VM phenotypes. In an attempt to perform an unbiased analysis of disease phenotypes associated with *ACTG2* variants, we performed gene variant extraction on the whole rare disease cohort within the Genomics England 100,000 disease project. We found 37 missense carriers (an *AF* < 1% in any gnomAD population and a CADD score > 20) and 164 synonymous variant carriers (with an *AF* < 1% in any gnomAD population). The phenotypes of these individuals with *ACTG2* variants (either rare and deleterious missense or synonymous as a control group) were analysed for associated ICD10 terms. In total, 405 statistical tests were performed, and statistical significance was adjusted for multiple testing with statistical significance *p* < 1.2 × 10^−4^ (0.05/405). Only ICD term Q64 ‘Other congenital malformations of urinary system’ reached phenome-wide statistical significance in the cohort, although several gastrointestinal phenotypes (potentially compatible with manifestations of visceral myopathy) reached nominal significance (Supplementary Table S6).

### Patients with rare KCNMA1 alleles

Heterozygous pathogenic variants in *KCNMA1* cause Liang-Wang syndrome (OMIM 618729), a severe neurological disorder that may include severe global developmental delay, craniofacial dysmorphism and visceral and connective tissue abnormalities [27]. Biallelic variant in *KCNMA1* is associated with cerebellar atrophy, developmental delay and seizures (OMIM 617643) [45]. *KCNMA1* encodes for a calcium-activated potassium channel and has a role in the modulation of vascular smooth muscle potassium channels [50]. Recently, a de novo heterozygous variant in *KCNMA1* (c.1123G > A; p.Gly375Arg) was reported in a child with absent abdominal musculature [3] which prompted us to examine our VM cohort for variants in this gene.

**Fig. 2:**
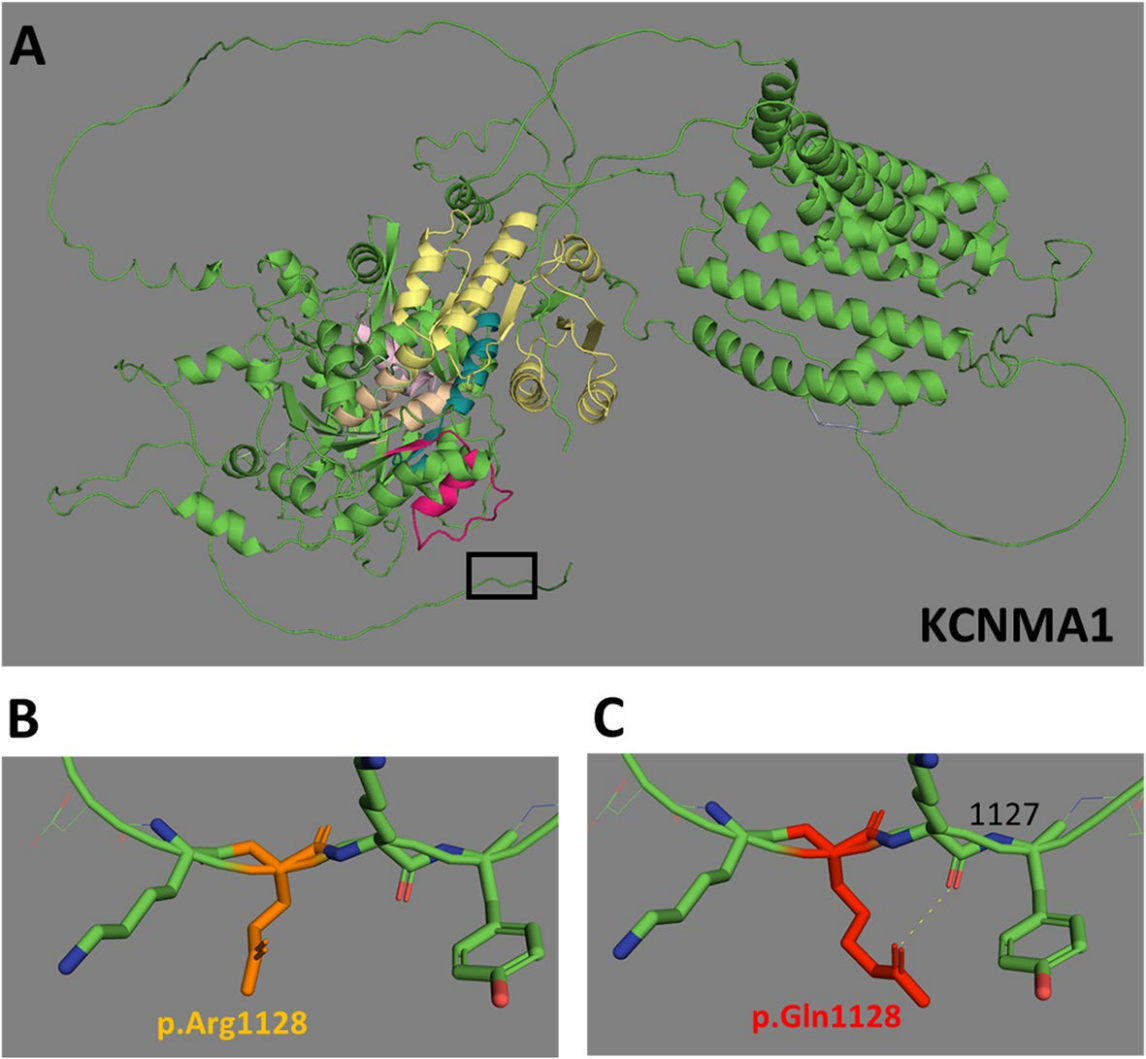
AlphaFold-2 models of *KCNMA1* missense variant. **A** Low-resolution model of KCNMA1 protein structure (NP_002238.2) with region containing amino acid position 1128 boxed. **B** Wild-type KCNMA1 (NP_002238.2) p.(Arg1128) and C. missense KCNMA1 p.(Arg1128Gln), with likely bond to adjacent leucine (position 1127)

We identified one VM patient with early onset intestinal pseudo-obstruction, megacystis, constipation, feeding difficulties and gastrointestinal dysmotility with a heterozygous *KCNMA1* rare variant (Table 3 and Supplementary Fig. S3). This missense variant p.(Arg1128Gln) has a low SIFT score (0.07) and high PolyPhen score (0.991) and is classified as a VUS using the ACMG criteria. The unaffected father was wild type for the allele, and the mother (also affected with joint hypermobility, poor wound healing and constipation) had the same heterozygous variant, suggesting an autosomal dominant pattern of inheritance of a VM-related phenotype. This variant therefore remains an interesting and novel candidate variant for the VM phenotypes exhibited in this family. The allele frequency within the 100,000 genomes rare disease project was 0.00002. The gnomAD allele frequency, a ‘healthy control population’, is 0.000012, suggesting that either this allele is not fully penetrant or can cause subclinical phenotypes. For missense alleles in *KCNMA1*, gnomAD data shows a Z score of 5.06, indicating some intolerance to variation. Modelling of this variant showed a predicted new abnormal bond to the adjacent on lysine at position 1127 which provides some evidence to support its pathogenicity (Fig. 2).

**Table 3:** Patient phenotypes and *KCNMA1* alleles identified following WGS

Participant	Ethnicity	Phenotype	*KCNMA1* genetic variant	Amino acid change	ACMG classification	SIFT	PolyPhen-2	Allele frequency	Solved by GEL	Comments	Reference or novel
24 — female	White, British	Intestinal pseudo-obstruction, megacystis, constipation, feeding difficulties, gastrointestinal dysmotility	**c.3383G > A**	p. (Arg1128Gln)	Uncertain significance (1 point) PM2 supporting PP3 supporting BP1 supporting	Deleterious (0.02)	Probably damaging (0.999)	0.000012 (gnomAD)	*N*	rs200141207 Mother affected with same genotype; father unaffected, no genotype available	Novel

*KCNMA1* NM_002247.4 SIFT A value of between 0 and 0.05 is predicted to affect protein function. PolyPhen-2 value between 0.85 and 1.0 is confidently predicted to be damaging; values between 0.15 and 1.0 range are possibly damaging. Values between 0.0 and 0.15 are predicted to be benign

## Discussion

We present the first examination of the Genomics England rare disease database for the VM group of diseases, a diverse set of phenotypes with a wide spectrum of clinical severity. We identified known pathogenic and novel variants in *ACTG2* and *MYH11* likely accounting for a total of 12 cases of VM-related phenotypes*.* Also, in this set of known VM-causing genes, we identified several VUS that require further evaluation. In *KCNMA1*, a candidate gene for VM, we identified a novel missense variant that appeared to segregate with VM phenotypes in an autosomal dominant manner. This variant requires further investigation and validation however. For the genetically solved cases in *ACTG2*, all variants were rare heterozygous missense variants with high PolyPhen and low SIFT scores (i.e. predicted highly pathogenic) in patients with VM, with no other explanatory variants. The 9 *ACTG2* variants were also classed as pathogenic using ACMG criteria. For the genetically solved cases in *MYH11*, all 4 patients shared a heterozygous pathogenic frameshift mutation previously associated with VM [5] and intestinal pseudo-obstruction [9] in an autosomal dominant pattern.

The genome-wide variant burden test which was performed on the VM cohort identified *ACTG2* as the major gene implicated and is consistent with our gene panel findings for this cohort. As confirmatory evidence, *ACTG2* was also recently identified as the most strongly associated gene in patients with gastrointestinal disorders using a Bayesian genetic association method [10]. The extreme variability in disease phenotypes suggests that VM-related disorders are likely to be multifactorial disorders with genetic as well as epigenetic and environmental factors playing a role. Dissecting out each of these contributing factors will be important for the understanding of these conditions [14].

Unbiased phenotypic enrichment analysis did not suggest additional phenotypes for *ACTG2* beyond kidney and gastrointestinal tract involvement. This does not exclude completely that *ACTG2* variants could contribute to milder gastrointestinal phenotypes such as malabsorption and ileus, and further work in less severe disease cohorts is warranted.

This study’s strength lies in the systematic nature of the WGS database examination and the database itself. The Genomics England database is a large national database comprising ~ 85,000 patients with rare disease and/or cancer, all of whom have had WGS [43]. We have applied rigorous searches to this dataset to ensure we identify any potential patient with VM and any rare, potentially pathogenic variants in the genes associated with VM. The main weakness in this study is the small number of patients with VM in the Genomics England database (*n* = 76) and the difficulty in identifying these patients from the recorded HPO and phenotypic descriptors. A general weakness of any study taking advantage of Genomic England data is the lack of detailed phenotypic information with no direct access to the participants’ clinicians or their imaging data.

*ACTG2*, the leading cause of VM [2], was the most highly represented gene in our analysis, a result confirmed by our genome-wide variant burden test study within the Genomics England cohort. The extent of *ACTG2* variation in the context of VM has previously been described [2], and patients with arginine substitutions in particular suffer more severe phenotypes. Assia Batzir et al. have also compiled a list of all known *ACTG2* variants associated with VM, all are heterozygous missense variants [2]. With this wealth of supporting data concerning *ACTG2* variants in association with VM phenotype,s we recommend a change in the nomenclature for these patients to ‘autosomal dominant *ACTG2* visceral myopathy’.

We identified missense *ACTG2* variants that were not clearly pathogenic. The alleles p.(Arg96His) and p.(Asn129Ser)) were associated with CAKUT and recurrent UTI phenotypes, respectively, but may possibly represent milder *ACTG2* clinical phenotypes. A previously reported family, where a mother of two siblings with microcolon, did not have bladder/bowel involvement, but did have postpartum uterine atony hint at this phenotypic diversity. All three family members had the same heterozygous *ACTG2* variant p.(Arg178Cys) [2].

The second most common cause of VM we identified was *MYH11*. In the literature, these are predominantly autosomal recessive cases with biallelic variants; however, autosomal dominant forms have been reported [5, 9]. We identified four patients with variable clinical phenotypes of VM, who had the same heterozygous p.(Pro1940Hisfs*91) frameshift allele, suggesting this allele may be an autosomal dominant cause of VM. We also identified a patient with Prune Belly syndrome with a novel heterozygous variant in *MYH11* (p.(Lys1141Gln)), which is classified as a VUS. Unfortunately, this patient was a singleton, and no segregation data is therefore available, and further inference about the pathogenicity of this variant is premature. Functional studies are needed to determine if these potential novel variants we have identified are indeed pathogenic.

The overall solve rate of this diverse phenotypically heterogeneous cohort was low at around 9%. Many of the known VM-associated genes, including *ACTA2*, *MYLK*, *LMOD1*, *CHRM3*, *MYL9* and *FLNA* did not have variants that explained the patients’ phenotypes. This implies that for VM patients, there may be many more alternative underlying genetic causes to be identified. As we have shown, variants in *MYH11* and *KCNMA*1 represent a possible genetic cause for this disease phenotype, but other yet to be discovered contributory genes will need to be identified.

## Conclusions

VM phenotypes are diverse but include severe, life-threatening disorders caused by smooth muscle weakness in the bladder, bowel and uterus. We present molecular genetic variants including novel alleles in *ACTG2* and *MYH11* associated with VM phenotypes that allow a precise molecular diagnosis to be reached. In our VM cohort, *ACTG2* mutations were the leading cause of VM, and we recommend a nomenclature change to autosomal dominant *ACTG2* visceral myopathy for such cases.

## Supplementary Information

**Additional file 1: Figure S1.** IGV visualisation of genetic variants in *ACTG2*. **Figure S2.** IGV visualisation of genetic variants in *MYH11*. **Figure S3.** IGV visualisation of genetic variants in *KCNMA1*. **Table S1.** OMIM IDs and HPO Terms used for phenotypic searches. **Table S2.** Patient phenotypes and MYLK alleles identified following WGS. **Table S3.** Patient phenotypes and *CHRM3* alleles identified following WGS. **Table S4.** Patient phenotypes and *FLNA *alleles identified following WGS. **Table S5.** Genome wide variant burden test in a cohort of patients with visceral myopathy phenotypes. **Table S6.** Top 20 ICD phenotypes enriched in carriers of rare predicted pathogenic *ACTG2* missense variants (compared to carriers of rare synonymous variant carriers) in the Genomics England 100,000 Genomes project. 

## Data Availability

Data sharing is available through the Genomics England Research Consortium.
