# Supplementary material for: Use of whole genome sequencing to determine the genetic basis of visceral myopathies including Prune Belly syndrome
Source: J Rare Dis (Berlin). 2023 Jun 5;2(1):9. doi: 10.1007/s44162-023-00012-z (PMC10241726; doi:10.1007/s44162-023-00012-z)
Supplement: Supplementary file 1 — Additional file 1: Figure S1. IGV visualisation of genetic variants in ACTG2. Figure S2. IGV visualisation of genetic variants in MYH11. Figure S3. IGV visualisation of genetic variants in KCNMA1. Table S1. OMIM IDs and HPO Terms used for phenotypic searches. Table S2. Patient phenotypes and MYLK alleles identified following WGS. Table S3. Patient phenotypes and CHRM3 alleles identified following WGS. Table S4. Patient phenotypes and FLNA alleles identified following WGS. Table S5. Genome wide variant burden test in a cohort of patients with visceral myopathy phenotypes. Table S6. Top 20 ICD phenotypes enriched in carriers of rare predicted pathogenic ACTG2 missense variants (compared to carriers of rare synonymous variant carriers) in the Genomics England 100,000 Genomes project. [file 44162_2023_12_MOESM1_ESM.pdf]

Supplementary data

Use of whole genome sequencing to determine the genetic basis of visceral myopathies including Prune Belly syndrome

Robert M Geraghty[1,2], Sarah Orr[2], Eric Olinger[2], Ruxandra Neatu[2], Miguel Barroso-Gil[2], Holly Mabillard[2], Genomics England Research Consortium, Ian Wilson[3] and John A. Sayer[1,2,4]

We acknowledge members of The Genomics England Research Consortium:

John C. Ambrose1 ; Prabhu Arumugam1 ; Roel Bevers1 ; Marta Bleda1 ; Freya Boardman-Pretty1,2; Christopher R. Boustred1 ; Helen Brittain1 ; Mark J. Caulfield1,2; Georgia C. Chan1 ; Greg Elgar1,2; Tom Fowler1 ; Adam Giess1 ; Angela Hamblin1 ; Shirley Henderson1,2; Tim J. P. Hubbard1 ; Rob Jackson1 ; Louise J. Jones1,2; Dalia Kasperaviciute1,2; Melis Kayikci1 ; Athanasios Kousathanas1 ; Lea Lahnstein1 ; Sarah E. A. Leigh1 ; Ivonne U. S. Leong1 ; Javier F. Lopez1 ; Fiona Maleady-Crowe1 ; Meriel McEntagart1 ; Federico Minneci1 ; Loukas Moutsianas1,2; Michael Mueller1,2; Nirupa Murugaesu1 ; Anna C. Need1,2; Peter O'Donovan1 ; Chris A. Odhams1 ; Christine Patch1,2; Mariana Buongermino Pereira1 ; Daniel Perez-Gil1 ; John Pullinger1 ; Tahrima Rahim1 ; Augusto Rendon1 ; Tim Rogers1 ; Kevin Savage1 ; Kushmita Sawant1 ; Richard H. Scott1 ; Afshan Siddiq1 ; Alexander Sieghart1 ; Samuel C. Smith1 ; Alona Sosinsky1,2; Alexander Stuckey1 ; Mélanie Tanguy1 ; Ana Lisa Taylor Tavares1 ; Ellen R. A. Thomas1,2; Simon R. Thompson1 ; Arianna Tucci1,2; Matthew J. Welland1 ; Eleanor Williams1 ; Katarzyna Witkowska1,2; Suzanne M. Wood1,2.

1. Genomics England, London, UK; 2. William Harvey Research Institute, Queen Mary University of London, London, EC1M 6BQ, UK

Supplementary Table S1. OMIM IDs and HPO Terms used for phenotypic searches

Condition	OMIM ID	HPO terms
Prune Belly syndrome (PBS)	100100	HP:0003270; HP:0004392
Megacystis-Microcolon Hypoperistalsis syndrome (MMIHS)	619362, 249210, 619365, 619431, 619351	HP:0000021; HP:0002838; HP:0004388; HP:0010956; HP:0100771
Chronic Intestinal Pseudo-obstruction (CIPO)	3000048	HP:0004389; HP:0005249

Supplementary Table S2. Patient phenotypes and *MYLK* alleles identified following WGS

Participant	Phenotype	*MYLK* Nucleotide change	*MYLK* Amino Acid Change	ACMG Classification	SIFT	PolyPhen-2	Allele Frequency	Solved by GEL	Comments	Reference
17 – Female	Chronic constipation, neurogenic bowel, urinary incontinence, intestinal pseudo-obstruction	**c.4185C>T**	p.(=)	Likely benign (-4 points)	N/A	N/A	$1.99x10^{-5}$ (gnomAD)	N	Splice AI result: low impact	Novel
18 – Female	Intestinal pseudo-obstruction, constipation, myopathy, gastrointestinal dysmotility	**c.3611G>A**	p.(Arg1204Gln)	Likely benign (-1 point)	Deleterious (0.03)	Possibly damaging (0.668)	$2.57x10^{-5}$ (gnomAD)	N	rs142421063 - VUS	Novel

MYLK NM_053025.4
SIFT values of between 0 and 0.05 is predicted to affect protein function. PolyPhen-2 values between 0.85 to 1.0 are confidently predicted to be damaging, values between 0.15 to 1.0 range are possibly damaging. Values between 0.0 to 0.15 are predicted to be benign.

Supplementary Table S3. Patient phenotypes and *CHRM3* alleles identified following WGS

Participant	Phenotype	*CHRM3* Nucleotide change	*CHRM3* Amino Acid Change	ACMG classification	SIFT	PolyPhen-2	Allele Freq	Solved by GEL	Comments	Reference
20 - Male	Intestinal pseudo-obstruction, megacystis, constipation	**c.1461G>T**	p.(Lys487Asn)	Uncertain Significance (0 points)	Deleterious (0)	Probably damaging (0.995)	$3.98x10^{-6}$ (gnomAD)	N	rs1475331331 - VUS	Novel

CHRM3 NM_000740.4
SIFT values of between 0 and 0.05 is predicted to affect protein function. PolyPhen-2 values between 0.85 to 1.0 are confidently predicted to be damaging, values between 0.15 to 1.0 range are possibly damaging. Values between 0.0 to 0.15 are predicted to be benign.

Supplementary Table S4. Patient phenotypes and *FLNA* alleles identified following WGS

Participant	Phenotype	*FLNA* Nucleotide change	FLNA Amino Acid Change	ACMG Classification	SIFT	PolyPhen-2	Allele Frequency	Solved by GEL	Comments	Reference
21 - Male	Megacystis, congenital megaureter	**c.1884C>T**	p.(=)	N/A	N/A	N/A	N/A	N		Novel
21 - Male	Megacystis, congenital megaureter	**c.5741-8C>T**	N/A (splice region variant)	N/A	N/A	N/A	N/A	N	Splice AI result: low impact	Novel

21 - Male	Megacystis, congenital megaureter	**c.5289+4C>T**	N/A (splice region variant)	Benign (-10 points)	N/A	N/A	N/A	N	Splice AI result: low impact	Novel

FLNA NM_001456.4
SIFT values of between 0 and 0.05 is predicted to affect protein function. PolyPhen-2 values between 0.85 to 1.0 are confidently predicted to be damaging, values between 0.15 to 1.0 range are possibly damaging. Values between 0.0 to 0.15 are predicted to be benign.

Supplementary Table S5. Genome wide variant burden test in a cohort of patients with visceral myopathy phenotypes

Gene	Position	Cases with qualifying variants	Cases without qualifying variants	Controls with qualifying variants	Controls without qualifying variants	p-value
ACTG2	chr2:73892314-73910865	7	56	2	916	$1.1x10^{-7}$

Supplementary Table S6. Top 20 ICD phenotypes enriched in carriers of rare predicted pathogenic *ACTG2* missense variants (compared to carriers of rare synonymous variant carriers) in the Genomics England 100,000 Genomes project

ICD10	Prev. mis.	Prev. syn.	RR	Fisher's exact test	Phenotype (ICD)
**Q64**	**0.19**	**0.00**	**na**	**4.35E-06**	**Other congenital malformations of urinary system**
Q62	0.14	0.01	22.16	8.69E-04	Congenital obstructive defects of renal pelvis and congenital malformations of ureter
K90	0.16	0.02	8.86	1.48E-03	Intestinal malabsorption
Q43	0.16	0.02	8.81	1.48E-03	Other congenital malformations of intestine
K91	0.19	0.03	6.21	1.66E-03	Postprocedural disorders of digestive system, not elsewhere classified
Z93	0.27	0.07	3.69	1.77E-03	Artificial opening status
R14	0.14	0.01	11.08	2.63E-03	Flatulence and related conditions
Z04	0.14	0.01	11.08	2.63E-03	Examination and observation for other reasons
B96	0.27	0.08	3.41	2.71E-03	Other specified bacterial agents as the cause of diseases classified to other chapters
J06	0.27	0.08	3.41	2.71E-03	Acute upper respiratory infections of multiple and unspecified sites
Y83	0.30	0.10	3.05	2.98E-03	Surgical operation and other surgical procedures as the cause of abnormal reaction of the patient, or of later complication, without mention of misadventure at the time of the procedure
K56	0.19	0.04	5.17	3.11E-03	Paralytic ileus and intestinal obstruction without hernia
E87	0.24	0.07	3.63	3.52E-03	Other disorders of fluid, electrolyte and acid-base balance
R10	0.35	0.14	2.51	4.13E-03	Abdominal and pelvic pain
R11	0.32	0.12	2.66	4.27E-03	Nausea and vomiting
K52	0.22	0.05	3.94	4.44E-03	Other noninfective gastroenteritis and colitis
A41	0.19	0.04	4.43	5.36E-03	Other sepsis
H92	0.08	0.00	na	5.83E-03	Otalgia and effusion of ear
Q52	0.08	0.00	na	5.83E-03	Other congenital malformations of female genitalia
T82	0.14	0.018	7.39	6.05E-03	Complications of cardiac and vascular prosthetic devices, implants and grafts

Prevalences of each ICD10 term that was encountered in missense carriers were calculated inside the missense group (Prev. Mis.) and the synonymous group (Prev. Syn.) and phenotype enrichment ratio (“relative risk”, RR) in missense group was calculated

Supplementary Figure S1. IGV visualisation of genetic variants in *ACTG2*

Participant	*ACTG2* Nucleotide change	*ACTG2* Amino Acid Change	WGS variant visualised on IGV
1	c.119G>A	p.(Arg40His)	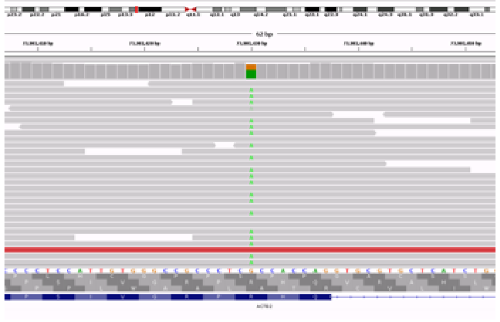
2	c.442C>T	p.(Arg148Cys)	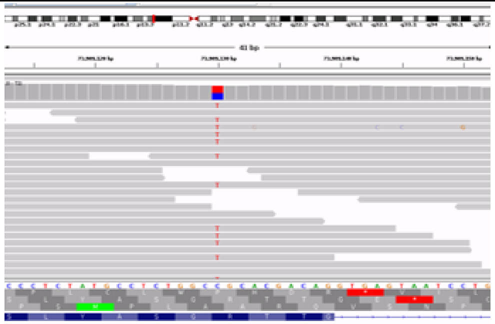
3	c.533G>T	p.(Arg178Leu)	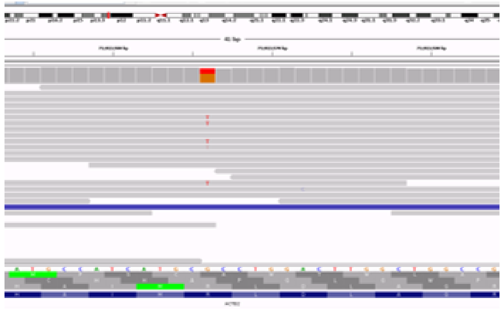

Participant	*ACTG2* Nucleotide change	*ACTG2* Amino Acid Change	WGS variant visualised on IGV
7	c.226A>T	p.(Ile76Phe)	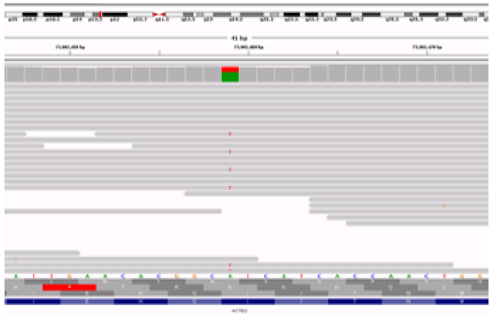
8	c.287G>A	p.(Arg96His)	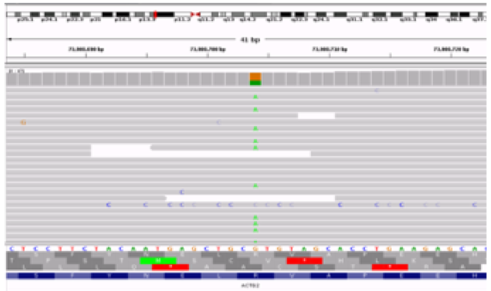
9	c.338C>T	p.(Pro113Leu)	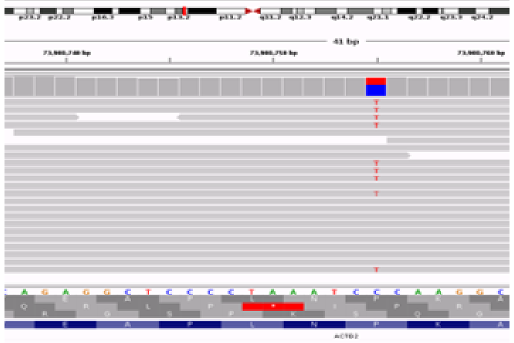

4	**c.769C>T**	p.(Arg257Cys)	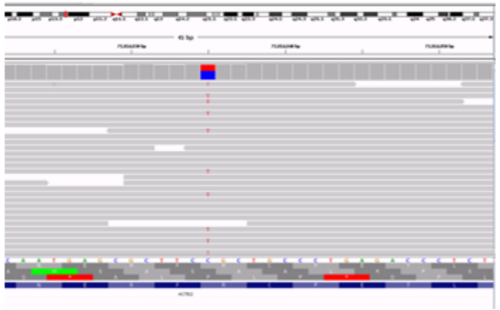
5	**c.770G>A**	p.(Arg257His)	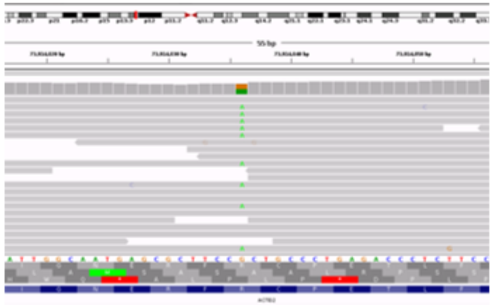
6 – Male	**c.770G>A**	p.(Arg257His)	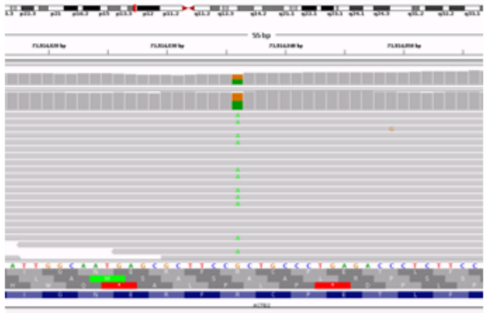

10	**c.386A>G**	p.(Asn129Ser)	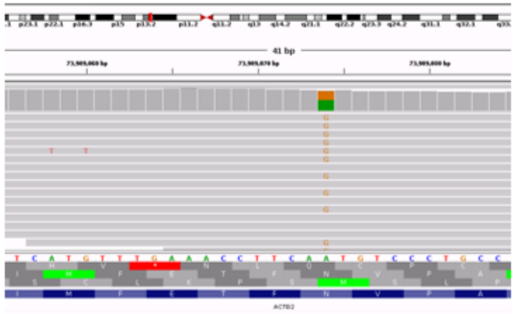
11 – Male	**c.850A>G**	p.(Met284Val)	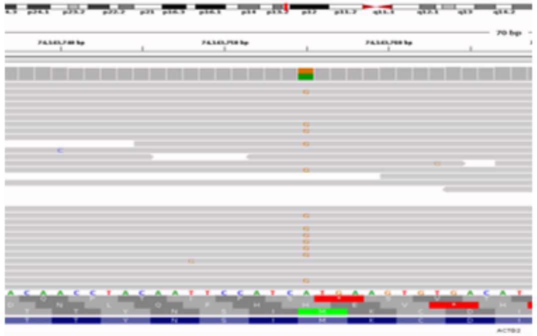

Supplementary Figure S2. IGV visualisation of genetic variants in *MYH11*

Participant	*MYH11* Nucleotide change	*MYH11* Amino Acid Change	WGS variant visualised on IGV
12	c.5819del	p.(Pro1940Hisfs*91)	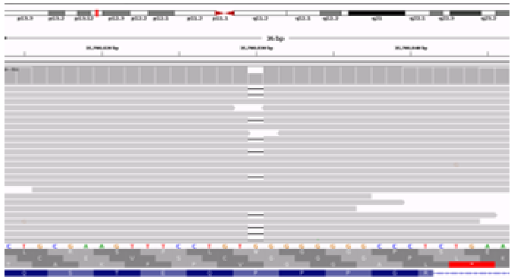
13	c.5819del	p.(Pro1940Hisfs*91)	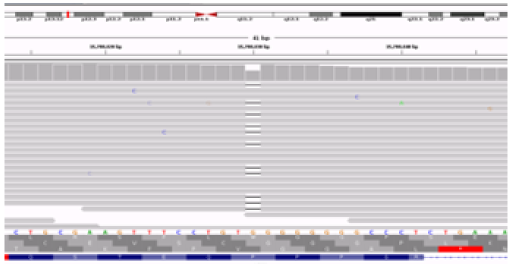
14	c.5819del	p.(Pro1940Hisfs*91)	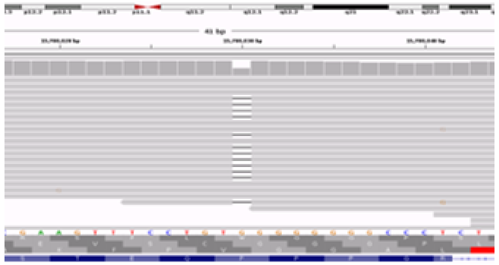
15	c.5819del	p.(Pro1940Hisfs*91)	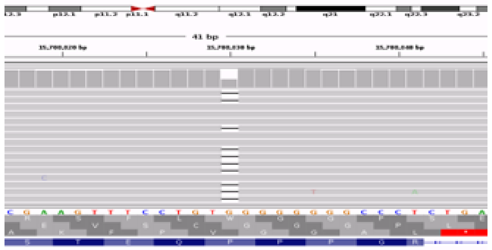

Supplementary Figure S3. IGV visualisation of genetic variants in *KCNMA1*

Participant	*KCNMA1* Nucleotide change	*KCNMA1* Amino Acid Change	WGS variant visualised on IGV
24	c.3383G>A	p.(Arg1128Gln)	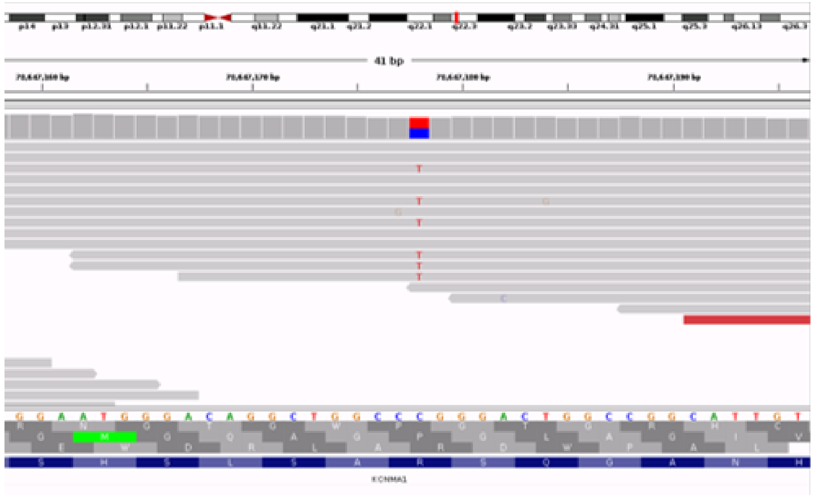
